# Supplementary material for: Adverse drug effects among students following mass de-worming exercise involving administration of Praziquantel and Albendazole in KEEA Municipality, Ghana
Source: PLoS Negl Trop Dis. 2022 Sep 12;16(9):e0010680. doi: 10.1371/journal.pntd.0010680 (PMC9499283; doi:10.1371/journal.pntd.0010680)
Supplement: Attachment — Submitted filename: Reviewer report.pdf [file pntd.0010680.s002.pdf]

Dear authors thank you for your work and coming up with such an interesting research area "Adverse drug effects among school pupils following mass de-worming exercise involving administration of praziquantel and albendazole in Ghana where such data are limited"**.**

Here are my comments and questions in the order of your document.

1. Abstract: result; line 25-26: "Commonly reported adverse effects were headaches, Abdominal pains", and somewhere in the document "headache, abdominal pain, line 216-219, page 11", consistency of word or idea is important.
2. Introduction: "The major setback in using these drugs in school pupils is the associated adverse effects which are normally mild and transient and also subside rapidly". How this could be a problem if the reported side effects are mild and subside rapidly? What is the need to monitor and record these side effects which are not troublesome to the pupils? Better to change the statement and show any possible impact of these reported side effects on the daily activities of pupils, such as school adherence.
3. Ethical consideration: is this the journal's policy to give "Ethical consideration" at the beginning of the method section?
4. Under "Methods", the study design and duration of the study are not stated?
5. Under "Methods; Study Population"; It was stated that "A total of 598 school pupils aged between 5 - 19 years in both Primary and Junior High School and permanently residing in the 4 communities in KEEA Municipal were randomly selected for the study". But, for the other researcher/ reader it will not be clear how the four primary and junior high schools residing in the 4 communities were selected and how the 598 samples were proportionally allocated to these four primary and junior high schools. From how many pupils 598 pupils were selected?
6. There was a terminology without definition for your research (Adverse drug effect); as a reader of your paper, what is the definition of adverse drug effect per your study? In the method parts, try to give the definitions?. In addition, the authors used side effects and adverse effects interchangeably although they have different meanings, need justification?
7. Under "Safety Monitoring and Adverse Events Management part" it was stated, A study physician was available at the district hospital to attend to emergencies, needs clarification.

8. Under “Result section, Line 217-219, page11”, it was stated as “Multiple effects were reported among some of the pupils. Some complained of more than two adverse effects which include headache, diarrhea, and increased body temperature; abdominal pain, headache, and increased body temperature (Figure 4)” which is difficult to understand. Clarification is required on these statements.
9. Conclusion: Line 297-298, “The study showed that over one-third of school children recruited into the study experienced one or two forms of adverse effects” But under the result section, there were children with more than two reported side effects, APHIT: abdominal pains, headache and increased temperature, HDIT: headache, diarrhea and increased temperature Line 226-227, page 12. The conclusion of the study should be supported by the results reported in the manuscript.
10. Under conclusion “Line 297-298, page 16” Although these adverse effects were transient and mild, they should not be overlooked during future mass drug administration. There was a terminology without definition for your research (mild, moderate, severe adverse drug effect); as a reader of your paper, what is the definition of mild adverse drug effect per your study? In the method parts, try to give the definitions and justify how the authors handle severe adverse drug events that occur during the study (if any)?
