# Supplementary material for: Adverse drug effects among students following mass de-worming exercise involving administration of Praziquantel and Albendazole in KEEA Municipality, Ghana
Source: PLoS Negl Trop Dis. 2022 Sep 12;16(9):e0010680. doi: 10.1371/journal.pntd.0010680 (PMC9499283; doi:10.1371/journal.pntd.0010680)
Supplement: Attachment — Submitted filename: Comments to the author for PNTD-D-21-01133.pdf [file pntd.0010680.s003.pdf]

The paper explored the incidence and type of adverse drug effects or events (ADE) among school pupils in KEEA Municipality of Ghana after mass drug administration in an effort to fight lymphatic filariasis and schistosomiasis. The study presented significant findings about the incidence of ADE. However, I would like to point out multiple points that needs to be resolved before making this manuscript good for the readers:

- <u>Main point:</u>
  - I believe the authors in this study were not investigating all adverse events (AE) that occurred during MDA, instead they were investigating only adverse drug events (ADE). Therefore, they should use the term adverse drug effect or event (ADE) instead of the general term "adverse events (AE)". You need to revise the difference between these terms to select the term that fir your objective. After deciding on the correct term for your objective based on your methods, you need to apply this correction to all parts of this manuscript and you may use the abbreviation throughout the manuscript.
- In the abstract,
  - In page 1, line 27 -28: in the sentence "Incidence of adverse drug effects among males was not significantly (51% versus 49%, $p > 0.05$) different from that of females.", the percentages of ADE for male and female are more than one-third that was indicated at the beginning of the results section of the abstract. This could be a typo error or otherwise you need to revise you results.
  - In page 2, line 32: I suggest that you choose a word that better suites the sentence than "institute". You may consider words such as: develop or create.
- The introduction is very comprehensive and
  - In page 2, line 53-55: in the sentence "It has been estimated that more than 200 million people …" is this number annually? Consider revising this sentence for clarification.
  - In page 3, line 83-84: the sentence "Similarly, MDA compliance has been reported to reduce prevalence of LF [14], Egypt [15] and Tanzania [16]." I was not able to understand this sentence, please revise the writing of this sentence.
- Your methods section although was almost complete, it was not organized and needs to be reorganized and completed to simplify the understanding of this section:
  - Start your methods section with the first paragraph which could be titled "Study design and population" where you indicate the design for the study and restating the purpose of the study. In this paragraph you will briefly indicate the site or location where the study was conducted, how the data were collected, your targeted population (by age and level in school), the medication that were

administered, and how did you do the monitoring. This paragraph will help you introduce the reader to your following sections of the methods. Then, your “Ethical Consideration” section will be kept as is. The following two sections should be the “Study Area” and “Study Population”. The following sections should be “Drug Administration”, “Interview and Measurements”, “Safety Monitoring and Adverse Drug Events Management”, and “Statistical Analysis”.

- In page 5, in the “Study Population” section: Reorganize this section to start with the justification why these studies were selected (currently the last sentence in the paragraph). Then, indicate the actual number of primary and junior high schools where data were collected before listing these schools’ names. Also, the number of students should not be mentioned in here as this should part of your results.
- In page 5, in the “Drug Administration” section: I didn’t understand if all pupils should receive both medications or either one and why there would be discrepancy among the pupils in receiving the medication from the same MDA program. I saw that in the results as well, some pupil received both medications and others didn’t, you need to describe this in your methods. Also, this needs to be described in more details on how these medications were administered by the MDA program? Administered at home or in school, both tablets at the same time or a tablet on each day or ….? This would make a difference in the interpretation of your results. This should be adjusted then this adjustment should be reflected on the rest of the manuscript.
- In page 5, in the “Interview and Measurements” section: you need to indicate when the actual data collection was conducted as it relates to the day of MDA in the school.
- In page 5, your “Statistical Analysis” section needs major revision and reorganization. You may start with the descriptive data analysis, followed by the inferential statistics and try to make it more specific. Then you need to provide your sample size calculation. Then, the level of significance for the study can be listed. Finally, you may end up by the statistical software that were used in your analysis.

- The results section:
  - In page 6, line 160: the sentence needs revision “A total of 598 Basic school pupils were recruited into the MDA program” this cannot be true. If you included all patients that received the medication and they total only 598 pupils then this is not an MDA. You need to revise this statement.
  - In the results section, you don’t restate the numbers from the tables you rather need to focus on the important number only.
  - Whenever you conduct inferential statistics, you need to interpret the results from these tests and report it whenever its significant. Otherwise, don’t conduct the analysis that you can not interpret its meaning. This was true for the results of the “Praziquantel only (Tablet)” in table 1, if you don’t know the interpretation of this analysis then just remove the p-values from the table as this has minimal to no value in your study and it is not your objective.

- In page 8, line 188-189: the use of “post drug administration” give the reader a false sense that there were pupils that had ADE before drug administration or that you have monitored a control group who didn’t receive medication.
- The comparison between male and female was not the purpose of the study but you kept comparing between them with no much value for the reader.

- General comments on the discussion section:
    - Due to the flaws in the analyses and presentation of the results, the authors were not able to construct informative discussion and conclusion section for the reader.
    - The limitation section should address all the actual limitations in the study. The efficacy of the medication was not your objective so it is not a limitation in your study.
- For the Tables and Figures:
    - Table 1:
        - you need to add the word “year” for the variable age group.
        - JHS: need to be defined in the caption for the table.
    - Figure 1:
        - Although not very necessary, you may keep it but adjust the caption for these figures “(A) Total post-treatment adverse effects.” There is not pre-treatment adverse effects assessment, was there? “(B) Gender association with post-treatment adverse effects stratified by class of the participants.” This is just the proportion of the incidence of ADE stratified by gender and school level.
        - Also, the vertical line is not really representing the data in the figure. This figure can be revised for better representation and simplified interpretation. Also, if any inferential statistics were conducted on these data then the results (p-value) should be presented on the figure.
- General points:
    - In regards to the use of abbreviation:
        - Whenever you introduce an abbreviation, you should use it throughout the manuscript and stop using the main term. This was very common for the “MDA” abbreviation.
        - Try not to introduce an abbreviation that you will not use again in the manuscript, to avoid any confusion for the readers.
        - In page 8, line 191: JHS was used without previous definition in text. You need to define it first then use the abbreviation onward.
    - In page 4, line 4: the word “Majority” should be with small letter.

- In page 5, lines 132-137: in the sentence "Each pupil was given one (1) tablet of albendazole …" we know that one is (1), you don't have to restate it into parenthesis. Just keep "one" in this case and the same is true in the following sentences. Unless you are going to have "half" then you need to state the number in wording "one, two, three, four, and five".
- In page 5, lines 134-137: the word "Tablet" should be with small letter.
- In page 5 lines 23-129: the words "primary", "junior high", and "school" does not have to be started with capital letters. Only the name of the school should be started with Capital letter. This was the case in the result section as well, so you need to adjust it everywhere in your manuscript.
- In page 6, line 160: no need for the ward "Basic".
- In page 6, line 162: "Body mass index (BMI)" this should be body mass index (BMI) but since it was mentioned in the methods then you may use the abbreviation directly.
- In page 6, line 163-165: don't start sentences with numbers, try to rephrase these sentences "39.97% of the pupils received only praziquantel tablets ranging from 1 tablet to 5 based on their weight, age and height. 527 of these school children ate before drug intake".
- In page 11, line 217 and 219-220: you referred to Figure 2 and 4 in an inadequate way which was misleading for the reader. Specially that there is no Figure 4 in your manuscript.

Overall, the authors had a good idea, methodology, and data, but didn't analyze or present their rich data in a way that would make it beneficial for the readers. They made multiple analyses that were not necessary and presented their data in a way that were confusing rather than informing for the readers. The impact of the insufficiently described and written "Statistical Analysis" section in their methods lead to inadequate description of their results section. This was true for almost all subsections in the results (3.2., 3.3., 3.4., 3.5., and 3.7. [no 3.6. section was reported]) as well as Tables 1, 2, 3, 4, and 5 and Figures 1B and 2B. They may have much better results section that is more informing and clearly described for the readers. However, I recommend that they consult with someone specialized in epidemiology or public health to select the appropriate data analyses and interpretation for their data to better present the rich and valuable data that they have.
