# Supplementary material for: Adverse drug effects among students following mass de-worming exercise involving administration of Praziquantel and Albendazole in KEEA Municipality, Ghana
Source: PLoS Negl Trop Dis. 2022 Sep 12;16(9):e0010680. doi: 10.1371/journal.pntd.0010680 (PMC9499283; doi:10.1371/journal.pntd.0010680)
Supplement: Attachment — Submitted filename: PLoS ONE NTD.docx [file pntd.0010680.s001.docx]

Thank you for giving me the opportunity to review this manuscript. This is a very well written and easy to follow manuscript. I really liked the study findings and how they are reported. I would like to congratulate the authors on conducting this study in one of our key school going students in Low and middle income countries (LMIC) such as Ghana and raising much-needed voice.

I have some suggestions and queries that will help improve the quality of the manuscript. I am sure you will not be disappointed by the end product once these changes have been made.

**Abstract:** The abstract is a little bit confusing. Please clearly state what do you want your readers to know and what is the key message in your abstract. For example, what do you want Disease Control Unit of the Ghana Health Service institute monitoring ADRs?

Also needs to be change, school pupils can be changed into school going students?

**Introduction:** Is it appropriate to discuss the significance of the study? What is the study's rationale? Previous literature from Ghana and school going students from other countries, if such data is available. Please highlight in the introduction part.

Please utilise current references; for example, references 3, 5, and 7 were published in 1997 and should be updated.

3. R. J. Stoltzfus, M. Albonico, J. M. Tielsch, H. M. Chwaya and L. Savioli, "School-based 335 deworming program yields small improvement in growth of zanzibari school children after one 336 year," The Journal of Nutrition, vol. 127, no. 11, pp. 2187-2193, 1997.

5. P. K. Das, K. D. Ramaiah, D. J. Augustin and A. Kumar, "Towards elimination of lymphatic 342 filariasis in india," Trends in parasitology, vol. 17, no. 10, pp. 457-460, 2001.

7. N. De Silva, H. Guyatt and D. Bundy, "Morbidity and mortality due to ascaris-induced 346 intestinal obstruction," Transactions of the Royal Society of Tropical Medicine and Hygiene, vol. 347 91, no. 1, pp. 31-36, 1997.

**Methods**
Excellent methodology; however, authors should include the study protocol if there any use for the data collection, how the interview conducted and what are the questions asked? However, it is only a suggestion that authors include a supplementary file or an appendix.

2.1. Ethical Consideration

“The study was approved by the Diseases Control Unit of Ghana Health Service who were 106 undertaking the mass drug administration exercise at the schools and Ghana Education Service 107 (Central Region)”

Please mentioned ethical approval number with year

All school pupils were invited to participate….please update people to students throughout the manuscript.

**Results:** I do not have any comments for this section.

**Discussion:**

Where is the study strength? as the limitations shows separately, make it as separate heading.

**References:** Please review formatting of references - again particularly for consistency with Journal title.

Please ensure that someone independent and highly proficient in written English has thoroughly checked/edited your revised submission.
