# Supplementary material for: Adverse drug effects among students following mass de-worming exercise involving administration of Praziquantel and Albendazole in KEEA Municipality, Ghana
Source: PLoS Negl Trop Dis. 2022 Sep 12;16(9):e0010680. doi: 10.1371/journal.pntd.0010680 (PMC9499283; doi:10.1371/journal.pntd.0010680)
Supplement: Attachment — Submitted filename: RESPONSE LETTER.docx [file pntd.0010680.s004.docx]

Dear Editor,

We acknowledge receipt of the reviewer comments. We found the comments to be very useful and therefore we have addressed all the comments to the best of our ability to improve the revised manuscript. For ease of perusal, we have highlighted all the corrections RED in the revised manuscript. We hope the improvements made in the revised manuscript will receive favorable response. Thank you.

Alex Boye (Ph.D)
