# Supplementary material for: Adverse drug effects among students following mass de-worming exercise involving administration of Praziquantel and Albendazole in KEEA Municipality, Ghana
Source: PLoS Negl Trop Dis. 2022 Sep 12;16(9):e0010680. doi: 10.1371/journal.pntd.0010680 (PMC9499283; doi:10.1371/journal.pntd.0010680)
Supplement: Attachment — Submitted filename: Comments to the Editor and the author for PNTD-D-21-01133 R1 to authors.docx [file pntd.0010680.s005.docx]

The paper explored the incidence and type of adverse drug effects or events (ADE) among school pupils in KEEA Municipality of Ghana after mass drug administration in an effort to fight lymphatic filariasis and schistosomiasis. The study presented significant findings about the incidence of ADE. Although the authors’ revision has improved the manuscript significantly, I would like to point out few points that needs to be addressed to make this manuscript good for readers:

- **Abstract**

Although this section is informative, I believe it is too much for an abstract specially in the background section. Whereas the methodology section can benefit from short description for the statistical part.

- **Introduction**

The introduction is much informative and has a better flow now and I enjoyed reading it and learning about the topic again. However, I had two minor comments about it:

- The paragraph that start with “Albendazole (ADZ), praziquantel (PZQT),…” is very lengthy and should be separated into two paragraphs at the middle.
- The last sentence in the introduction section seems like a conclusion for me which make is in the wrong location and should be removed. Instead, you can indicate the expected impact of this research without highlighting your findings.
- **Methods**

Very much improved in term of clarity and organization.

- **Results**

Your results section is much improved and became more meaningful.

- Note: avoid saying “students used for the study ….” You can say “students included in the study …” or “students participated in the study…”.
- **Discussion**

The discussion section needs major revision to be informative and organized. As it is currently written it is confusing and difficult to follow.

- The beginning of the first paragraph (298 - 305) is good and needed but it should have been added to the last paragraph of the introduction section.
- The last section of the first paragraph (308 – 312) is not related to the study objective and I think it is not necessary in here. However, the doses used in the study may supplement that reported in the methodology section.
- Your major findings from the study may complete the first paragraph instead of the unnecessary added sentences whereas the following paragraphs can be used to compare your results to the previous studies or similar studies in other countries to put your results into perspective.
- In line 328, you stated the results for “elsewhere” what do you mean by that? This paragraph (third paragraph) needs to be fully revised as it is not connected to each other and need more details.
- In the paragraph starting with “From the present study, most of the students …” many parts of it does not look right. Thus, the paragraph need revision and rewriting as I thought age was a significant factor in your results. Revise your results and rewrite this paragraph.
- In line 356, the sentence starting with “What was more revealing was the fact …” is very confusing and difficult to understand.
- In line 364, the paragraph starting with “Pharmacokinetics and metabolism of drugs …” doesn’t seem to be related to your objective. You should either introduce the connection or remove the paragraph.
- In line 372, the paragraph starting with “In the present study, only food intake before MDA” the information in here is confusing and I am not sure where did it come from. Revise your results and rewrite this paragraph.
- In the limitation section, line 401 and the sentence “therefore delayed-ADEs before or after 24 hours”, your study should have captured the ADE within 24 hours. So, delayed ADEs after 24 hours is the limitation in here.
- Your conclusion in the text is limited compared to your conclusion in the abstract. Revise both for improvement.
- **For the Tables and Figures:**
  - *Table 1 and 3:*
    - JHS need to be defined in the caption for the table.
  - *Figures*
    - Your figures have very much improved.
- **General points:**

Most of these general points were previously indicated but was not addressed by the authors:

- In page 6, line 163: the word “Majority” and “Municipality” should be with small letter.
- In page 7, lines 177-183: in the sentence “Each student was given one (1) tablet of albendazole …” we know that one is (1), you don’t have to restate it into parenthesis. Just keep “one” in this case and the same is true in the following sentences.
- In page 7, lines 180-183: the word “Tablet” or “Tablets” should be with small letter.
- In page 6 lines 162-163: the words “primary”, “junior high”, and “school” does not have to be started with capital letters. Only the name of the school should be started with Capital letter. This was the case in the result section as well, so you need to adjust it everywhere in your manuscript.
- In page 9, line 224: “Body Mass Index (BMI)” this should be body mass index (BMI) but since it was mentioned in the methods then you should use the abbreviation directly.
- In page 15, line 305: “Neglected Tropical Diseases (NTDs)” was defined twice in the same paragraph.
- In page 15, line 306: What do you mean by basic schools? I think schools should be fine.
- The manuscript may benefit from a revision by language editing service to improve the writing and flow of the paragraphs.
- In page 16, line 346: “increase in body temperature (fever)” you don’t have to define fever. Everyone knows what fever means.

Overall, the study was very informing and highlighted some area that should be considered by decision makers, but the writing and the organization need major revision.
