# Supplementary material for: Adverse drug effects among students following mass de-worming exercise involving administration of Praziquantel and Albendazole in KEEA Municipality, Ghana
Source: PLoS Negl Trop Dis. 2022 Sep 12;16(9):e0010680. doi: 10.1371/journal.pntd.0010680 (PMC9499283; doi:10.1371/journal.pntd.0010680)
Supplement: Attachment — Submitted filename: RESPONSE LETER.docx [file pntd.0010680.s006.docx]

Dear Editor,

We acknowledge receipt of the reviewer comments. We found the comments to be very useful and therefore we have addressed all the comments to the best of our ability to improve the revised manuscript. For ease of perusal, we have highlighted all the corrections RED in the revised manuscript. We hope the improvements made in the revised manuscript will receive favorable response. Thank you.

**REVIEWER COMMENTS**

The paper explored the incidence and type of adverse drug effects or events (ADE) among school pupils in KEEA Municipality of Ghana after mass drug administration in an effort to fight lymphatic filariasis and schistosomiasis. The study presented significant findings about the incidence of ADE. Although the authors’ revision has improved the manuscript significantly, I would like to point out few points that needs to be addressed to make this manuscript good for readers:

- **Abstract**

Although this section is informative, I believe it is too much for an abstract specially in the background section. Whereas the methodology section can benefit from short description for the statistical part.

RESPONSE: We appreciate the importance of the comments. Accordingly, we have improved the abstract in the revised manuscript.

- **Introduction**

The introduction is much informative and has a better flow now and I enjoyed reading it and learning about the topic again. However, I had two minor comments about it:

- The paragraph that start with “Albendazole (ADZ), praziquantel (PZQT),…” is very lengthy and should be separated into two paragraphs at the middle.
- The last sentence in the introduction section seems like a conclusion for me which make is in the wrong location and should be removed. Instead, you can indicate the expected impact of this research without highlighting your findings.

RESPONSE: We appreciate the importance of the comments, accordingly, we have improved the paragraph that begins with “Albendazole” and the last sentence of the introduction in the revised manuscript.

- **Methods**

Very much improved in term of clarity and organization.

RESPONSE: We thank you for the commendation.

- **Results**

Your results section is much improved and became more meaningful.

- Note: avoid saying “students used for the study ….” You can say “students included in the study …” or “students participated in the study…”.

RESPONSE: We appreciate the importance of the comment and we have accordingly improved the results section in the revised manuscript.

- **Discussion**

The discussion section needs major revision to be informative and organized. As it is currently written it is confusing and difficult to follow.

RESPONSE: We appreciate the importance of the comments. We have improved the discussion per the comments in the revised manuscript.

- The beginning of the first paragraph (298 - 305) is good and needed but it should have been added to the last paragraph of the introduction section.

RESPONSE: Accordingly rectified in the revised manuscript.

- The last section of the first paragraph (308 – 312) is not related to the study objective and I think it is not necessary in here. However, the doses used in the study may supplement that reported in the methodology section.

RESPONSE: Accordingly rectified in the revised manuscript.

- Your major findings from the study may complete the first paragraph instead of the unnecessary added sentences whereas the following paragraphs can be used to compare your results to the previous studies or similar studies in other countries to put your results into perspective.

RESPONSE: Accordingly rectified in the revised manuscript.

- In line 328, you stated the results for “elsewhere” what do you mean by that? This paragraph (third paragraph) needs to be fully revised as it is not connected to each other and need more details.

RESPONSE: Accordingly rectified in the revised manuscript.

- In the paragraph starting with “From the present study, most of the students …” many parts of it does not look right. Thus, the paragraph need revision and rewriting as I thought age was a significant factor in your results. Revise your results and rewrite this paragraph.

RESPONSE: Accordingly rectified in the revised manuscript.

- In line 356, the sentence starting with “What was more revealing was the fact …” is very confusing and difficult to understand.

RESPONSE: Accordingly rectified in the revised manuscript.

- In line 364, the paragraph starting with “Pharmacokinetics and metabolism of drugs …” doesn’t seem to be related to your objective. You should either introduce the connection or remove the paragraph.

RESPONSE: Accordingly rectified in the revised manuscript.

- In line 372, the paragraph starting with “In the present study, only food intake before MDA” the information in here is confusing and I am not sure where did it come from. Revise your results and rewrite this paragraph.

RESPONSE: Accordingly rectified in the revised manuscript.

- In the limitation section, line 401 and the sentence “therefore delayed-ADEs before or after 24 hours”, your study should have captured the ADE within 24 hours. So, delayed ADEs after 24 hours is the limitation in here.

RESPONSE: Corrections effected as suggested.

- Your conclusion in the text is limited compared to your conclusion in the abstract. Revise both for improvement.

RESPONSE: Accordingly, we have improved the two in the revised manuscript.

- **For the Tables and Figures:**
  - *Table 1 and 3:*
    - JHS need to be defined in the caption for the table.

RESPONSE: Rectified accordingly.

- *Figures*
  - Your figures have very much improved.

RESPONSE: We thank you for the commendation.

- **General points:**

Most of these general points were previously indicated but was not addressed by the authors:

- In page 6, line 163: the word “Majority” and “Municipality” should be with small letter.

RESPONSE: Rectified accordingly in the revised manuscript.

- In page 7, lines 177-183: in the sentence “Each student was given one (1) tablet of albendazole …” we know that one is (1), you don’t have to restate it into parenthesis. Just keep “one” in this case and the same is true in the following sentences.

RESPONSE: Accordingly rectified in the revised manuscript.

- In page 7, lines 180-183: the word “Tablet” or “Tablets” should be with small letter.

RESPONSE: Accordingly rectified in the revised manuscript.

- In page 6 lines 162-163: the words “primary”, “junior high”, and “school” does not have to be started with capital letters. Only the name of the school should be started with Capital letter. This was the case in the result section as well, so you need to adjust it everywhere in your manuscript.

RESPONSE: Accordingly rectified in the revised manuscript.

- In page 9, line 224: “Body Mass Index (BMI)” this should be body mass index (BMI) but since it was mentioned in the methods then you should use the abbreviation directly.

RESPONSE: Accordingly rectified in the revised manuscript.

- In page 15, line 305: “Neglected Tropical Diseases (NTDs)” was defined twice in the same paragraph.

RESPONSE: Accordingly rectified in the revised manuscript.

- In page 15, line 306: What do you mean by basic schools? I think schools should be fine.

RESPONSE: Accordingly rectified in the revised manuscript.

- The manuscript may benefit from a revision by language editing service to improve the writing and flow of the paragraphs.

RESPONSE: Accordingly rectified in the revised manuscript.

- In page 16, line 346: “increase in body temperature (fever)” you don’t have to define fever. Everyone knows what fever means.

Overall, the study was very informing and highlighted some area that should be considered by decision makers, but the writing and the organization need major revision.

RESPONSE: we appreciate the comment. Accordingly, we have the revised manuscript.
