# Supplementary material for: Adverse drug effects among students following mass de-worming exercise involving administration of Praziquantel and Albendazole in KEEA Municipality, Ghana
Source: PLoS Negl Trop Dis. 2022 Sep 12;16(9):e0010680. doi: 10.1371/journal.pntd.0010680 (PMC9499283; doi:10.1371/journal.pntd.0010680)
Supplement: Attachment — Submitted filename: Comments to the author for PNTD-D-21-01133 R2 to authors.docx [file pntd.0010680.s008.docx]

The paper explored the incidence and type of adverse drug effects or events (ADE) among school pupils in KEEA Municipality of Ghana after mass drug administration in an effort to fight lymphatic filariasis and schistosomiasis. The study presented significant findings about the incidence of ADE. Although the authors’ revision has improved the manuscript significantly, I would like to point out minor points that needs to be addressed to make this manuscript good for readers:

- **Abstract, Introduction, Methods, and Results:**

These sections have very much improved from the first and second drafts.

- **Discussion and conclusion:**

The discussion and conclusion sections still week and needs revision or better rewetting of some parts to be informative and organized. As it is currently confusing, difficult to follow, and even underestimate the value of the your results. Here are some points for you to consider when revising this part:

- The first paragraph resembles your introduction, so it is redundant. you need to cut down on it and keep the purpose of the study, the objective, and merge it with your main findings (second paragraph).
- The third (line 317) and fourth (line 336) paragraphs are connoted to each other but combining them would make it a very lengthy paragraph. Thus, you need to make the third concise and connect it with the fourth.
- The fifth paragraph (line 343) is very important part of your study and you need to make it stronger with more evidence from the literature and better presentation and organization.
- In the paragraph starting with “In the present study ….” (line 354) this needs to be revised as it is very confusing and it is not clear what you are trying to say. Also, the part about the pharmacokinetic of the medication is still confusing and poorly explained in terms of how it is related; I still don’t see how it is related but you may want to keep it if you can better explain its relation to the study and how this can solve the problem in the study. This is a very confusing paragraph that needs rewriting after specifying the idea for the paragraph.
- The seventh paragraph (line 374) is important as well but it just seems like listing of comparisons without the existence of real discussion. This needs to be revised for improvement.
- In line 388 and later on at the sentences starting from “Pharmacokinetics …” this part seems to be out of track and in your study the food intake was not significant but you still emphasized it as an important factor. Can you explain why this is important? Then revise this section till the end of the paragraph or even make it in a separate paragraph.
- Your conclusion in the text is still limited compared to your conclusion in the abstract. Revise both again for improvement.
- **Minor points:**
  - Anthelminthic was spelled ant-helminthic in multiple times. This needs to be fixed throughout the manuscript.
  - In the title of table 3, you said “by students by participants” I think this is a typo mistake that needs to be fixed. Also, you stated that you have done “multivariate logistic analysis” when you actually done “multivariable logistic analysis”, these are not the same think and you can read about the difference. If this was stated in other places then you need to fix it as well.
  - GIT abbreviation was used then defined later on, this needs to be fixed. This issue occurred multiple times with multiple abbreviations including ADZ in the first page.
  - You tend to introduce abbreviations that you never use again (i.e., MBDZ), restate the full name of the abbreviation instead (i.e., NTD), or even reintroduce the abbreviation in other sections of the manuscript (i.e., ADE in the conclusion section and NTD in other part of the manuscript). Pay attention and use abbreviation as it is supposed to be used.

Overall, the study was very informing and highlighted some area that should be considered by decision makers, but the writing and the organization need revision.
