# Supplementary material for: Adverse drug effects among students following mass de-worming exercise involving administration of Praziquantel and Albendazole in KEEA Municipality, Ghana
Source: PLoS Negl Trop Dis. 2022 Sep 12;16(9):e0010680. doi: 10.1371/journal.pntd.0010680 (PMC9499283; doi:10.1371/journal.pntd.0010680)
Supplement: Attachment — Submitted filename: Reviewer Comments 24 5 22.docx [file pntd.0010680.s007.docx]

Dear Editor

Thank you for providing this opportunity to review the manuscript. In my opinion, the manuscript is not only poorly written the methodology is not appropriate. It will take too much time to mention all the drawbacks. Thus, I rejected this submission for publishing in this journal.

Thanking you
